# The Bone-Protective Effect of Genistein in the Animal Model of Bilateral Ovariectomy: Roles of Phytoestrogens and PTH/PTHR1 Against Post-Menopausal Osteoporosis

**DOI:** 10.3390/ijms13010056

**Published:** 2011-12-22

**Authors:** Qing Miao, Jing-Ge Li, Shan Miao, Nan Hu, Jin Zhang, Song Zhang, Yan-Hua Xie, Jian-Bo Wang, Si-Wang Wang

**Affiliations:** 1 Institute of Materia Medica, Fourth Military Medical University, Xi’an 710032, China; E-Mails: miaoqing@fmmu.edu.cn (Q.M.); lijingge@fmmu.edu.cn (J.-G.L.); miaoshan@fmmu.edu.cn (S.M.); zhangsong801101@163.com (S.Z.); yanhuaxie@gmail.com (Y.-H.X.); yyswjb@fmmu.edu.cn (J.-B.W.); 2 Institute of Stomatology, General Hospital of PLA, Beijing 100853, China; E-Mail: beyondstephen@vip.sina.com; 3 Department of Plastic Surgery, Xijing Hospital, Fourth Military Medical University, Xi’an 710032, China; E-Mail: jinzisay@fmmu.edu.cn

**Keywords:** genistein, phytoestrogen, osteoporosis, PTH, PTHR1

## Abstract

Genistein, a major phytoestrogen of soy, is considered a potential drug for the prevention and treatment of post-menopausal osteoporosis. Mounting evidence suggested a positive correlation between genistein consumption and bone health both *in vivo* and *in vitro*. Earlier studies have revealed that genistein acted as a natural estrogen analogue which activated estrogen receptor and exerted anti-osteoporotic effect. However, it remains unclear whether PTH, the most crucial hormone that regulates mineral homeostasis, participates in the process of genistein-mediated bone protection. In the present study, we compared the therapeutic effects between genistein and nilestriol and investigated whether PTH and its specific receptor PTHR1 altered in response to genistein-containing diet in the animal model of ovariectomy. Our results showed that genistein administration significantly improved femoral mechanical properties and alleviates femoral turnover. Genistein at all doses (4.5 mg/kg, 9.0 mg/kg and 18.0 mg/kg per day, respectively) exerted improved bending strength and b-ALP limiting effects than nilestriol in the present study. However, genistein administration did not exert superior effects on bone protection than nilestriol. We also observed circulating PTH restoration in ovariectomized rats receiving genistein at the dose of 18 mg/kg per day. Meanwhile, PTHR1 abnormalities were attenuated in the presence of genistein as confirmed by RT-PCR, Western blot and immunohistochemistry. These findings strongly support the idea that besides serving as an estrogen, genistein could interact with PTH/PTHR1, causing a superior mineral restoring effect than nilestriol on certain circumstance. In conclusion, our study reported for the first time that the anti-osteoporotic effect of genistein is partly PTH/PTHR1-dependent. Genistein might be a potential option in the prevention and treatment of post-menopausal osteoporosis with good tolerance, more clinical benefits and few undesirable side effects.

## 1. Introduction

Osteoporosis is a universal major public health problem which is characterized by low bone mass, deterioration of bone tissues and increased risk of fracture. The incidence of osteoporosis varies from country to country. Cummings and Melton noted that the risk of osteoporotic fracture in the USA was higher in urban than in rural areas [1]. The prevalence of osteoporosis based on bone density at the femoral neck was found to be 18~28% in women and 6~22% in men over the age of 50 years due to a reduction in endogenous oestrogen production [2]. Estrogen plays crucial roles in maintaining bone restoration, in particular for females. And estrogen hormone replacement therapy (HRT) has been suggested as the main therapeutic measure for prevention and treatment of post-menopausal osteoporosis for decades [3]. However, prolonged HRT therapy is not well accepted due to its potential to increase the incidences of breast cancer and cardiovascular events [4]. Therefore, it is urgent for the clinicians to develop alternative therapy with less undesirable side effects that can immensely reduce the need for drugs usage.

Although there are few studies indicate negative outcomes, phytoestrogens still raise great interests in recent years due to their clinical benefits in a variety of estrogen-dependent disorders [5]. There has been considerable interest towards the consumption dietary phytoestrogens and their potential benefits to alleviate menopausal symptoms, enhance post-menopausal bone health and reduce cancer risk [6]. Among phytoestrogens, isoflavones exerts the greatest advantage because of its abundance in food sources, notably soy products, and to their wide commercial availability as nutritional supplements. As for osteoporosis, the incidence of fracture has been analyzed among 24,403 post-menopausal Chinese women who underwent a soy protein or isoflavone diet for a 4.5-year period. The result showed a significant linear negative association between soy protein or isoflavone consumption (≥21 mg daily) and fracture risk [7]. Genistein (GEN) is the main soy isoflavone and it is freely absorbed from the intestine, and a large fraction is converted to the 7β-*O*-glucuronide as it crosses the brush border and ultimately enters the portal vein. Genistein has been extensively studied for its important hormonal properties [8]. Indeed, as suggested by the structural resemblance with the endogenous ligand estradiol, genistein shows potent estrogen-like properties both *in vivo* and *in vitro*. Abundant studies have demonstrated the bone-sparing effects of genistein in experimental animal models. Our previous studies have suggested genistein revealed bone sparing effects in ovariectomized rats [9,10]. Bitto *et al.* also showed that genistein restored better quality bone than alendronate, raloxifene, and estradiol [11]. Further studies demonstrated that genistein prevented and restored bone in animal models of secondary osteoporosis induced by steroids as well [12,13]. Recently, a well-controlled clinical trial revealed that 3 years of consecutive genistein administration at the dose of 54 mg/day significantly improves bone markers at a rate comparable with other standard therapies for osteoporosis in post-menopausal women [14]. An estimated average intake of genistein at 0.01–12 mg/day in Asian countries significantly reduces the incidence of post-menopausal osteoporosis, whereas genistein rich diet is rare in Western countries, which partly explain the lower prevalence of osteoporosis in Eastern countries [15–18].

However, besides mimicking estrogen properties, the underlying mechanisms of genistein-mediated bone-sparing effects are not fully understood. Parathyroid hormone (PTH) is a major regulator of ionized calcium and phosphate concentrations in the blood and extracellular fluids. Parathyroid hormone receptor 1 (PTHR1) is a specific receptor for PTH and it belongs to the G-protein coupled receptor family [19]. Upon activation in the presence of PTH, PTHR1 triggers calcium and phosphorus mobilization, which leads to osteogenesis and bone turnover. The primary target organs for PTH/PTHR1 are kidney and bone. In the kidney, PTH/PTHR1 stimulate reabsorption of calcium from renal tubules, stimulate 1,25-dihydroxycholecalciferol (1,25-(OH)_2_-VitD) synthesis, and prevent reabsorption of phosphate. In bone, PTH/PTHR1 mediate bone resorption by osteoclasts and reduce osteoblast proliferation, resulting in calcium liberation and decreased bone mass [20]. Interestingly, intermittent PTH administration has been found to increase bone density, improve skeletal architecture, enhance biomechanical strength, and reduce fracture risk [21–24]. Therefore, PTH or analogue and PTHR1 are potential novel targets for the therapeutic strategy for osteoporosis. But whether PTH/PTHR1 participates in the bone-sparing effect in response to genistein therapy remains unclear. In this regard, the aim of the present study is to explore the PTH/PTHR1 mechanisms of genistein-mediated bone protection in ovariectomized rats and to compare clinical beneficial effects between HRT and genistein.

## 2. Materials and Methods

### 2.1. Reagents

#### Medicine

**Figure 1:**
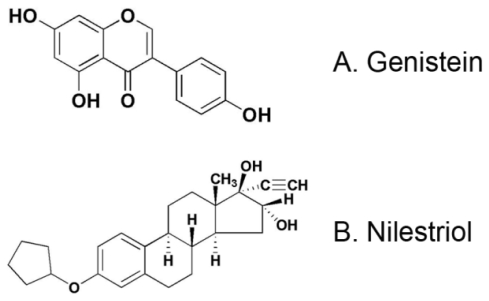
Chemical structure of genistein and nilestriol.

Genistein, 5,7-dihydroxy-3-(4-hydroxyphenyl)-4*H*-1-benzopyran-4-one (Figure 1A), and nilestriol, (8*R*,9*S*,13*S*,14*S*,16*R*,17*R*)-3-cyclopentyloxy-17-ethynyl-13-methyl-7,8,9,11,12,14,15,16-octahydro-6*H*-cyclopenta[α]phenanthrene-16,17-diol (Figure 1B), were purchased from Sigma (St. Louis, MO, USA).

#### Materials

Nine-week old female Sprague Dawley rats (250~300 g) obtained from the animal center of the Fourth Military Medical University were used for all experiments. This study conformed to the Guidelines for the Care and Use of Laboratory Animals published by the US National Institutes of Health (NIH publication No. 85-23, revised 1985). Rats were maintained under controlled conditions of room temperature (25 °C), relative humidity (50–80%) and illumination (12 h light, 12 h dark).

Trizol, RNA PCR kit and DNA Marker DL2000 were purchased from Takara (Otsu, Japan). Rabbit anti-PTHR1 antibody and HRP-conjugated goat anti-rabbit IgG were purchased from Abcam (Cambridge, MA, USA). HRP-conjugated sheep anti-mouse IgG was from GE Healthcare (Piscataway, NJ, USA). Rabbit anti-β actin antibody was obtained from Santa Cruz (Santa Cruz, CA, USA). PCR primer for PTHR1 was synthesized by Shanghai Bioengineering Corp. (Shanghai, China). b-ALP immunoassay kit was from Metra Biosystems Inc. (Santa Clara, CA, USA) and osteocalcin ELISA assay kit from Biomedical Technologies Inc. (Stoughton, MA, USA). PTH ELISA kit was from Immutopics Inc. (San Clemente, CA, USA). Chemiluminescence detection kit was obtained from Pierce (Rockford, IL, USA).

### 2.2. Experimental Protocols

The animal model of ovariectomy (OVX) was established as previously described. Briefly, rats were intraperitonally anaesthetized with 7% chloral hydrate (0.4 mL/100 g body weight). Bilateral ovariectomy was performed via a dorsal approach with a small midline dorsal skin incision. One week recovery after the surgery, rats were randomly assigned to one of the following six experimental groups. There were 20 rats in each group: (1) Sham group (Sham), animals received the operation while ovaries were not removed; (2) Ovariectomized group (OVX); (3) OVX + Nilestriol group (NIL): rats receive nilestriol diet at the dose of 1.5 mg/kg each week after OVX; (4) OVX + Low dose genistein group (GENL): rats receive genistein diet at the dose of 4.5 mg/kg per day after OVX; (5) OVX + Medial dose genistein group (GENM): rats receive genistein diet at the dose of 9.0 mg/kg per day after OVX; (6) OVX + High dose genistein group (GENH): rats receive genistein diet at the dose of 18.0 mg/kg per day after OVX. Diets were prepared by mixing the powdered with genistein or with nilestriol in the soy protein-free powdered semi-purified diet containing 0.97% calcium and 0.85% phosphorus. The rats were allowed to feed on regular diet only after finishing the mixture. After 12 weeks of treatment, the rats were euthanized by exsanguination under sodium pentobarbital anaesthesia after an overnight fasting. Blood samples were collected by cardiac puncture into heparinized tubes and immediately centrifuged (3500 g for 5 min at 4 °C). The serum was kept frozen at −20 °C until analysis.

### 2.3. Determination of Bone Mineral Density

Bone mineral density (BMD) was measured by dual energy X-ray absorptiometry (DEXA) (DPX-IQ 7040, Lunar Corp., Madison, WI, USA). Femurs and tibias densities on the left legs were measured at the end of the experiment.

### 2.4. Femoral Mechanical Testing

After collection, the length of the left femur and the mean width of the femoral diaphysis were measured using a caliper. Mechanical resistance was assessed by a three-point bending test using an Instron 8501 material testing system (Instron Corp., Canton, MA, USA). Samples were hydrated in physiological saline for 4 h prior to biomechanical testing. The diaphysis in each sample was tested to failure in a three-point bending test as we previously described [9,10]. Briefly, samples were placed on two supports spaced 15 mm apart to a pre-load of 1 Newton and then deformed at a rate of 1 mm/min until failure. The point of failure was defined as a successive drop in load greater than 5%.

### 2.5. Serum and Urine Mineral Analysis

Twenty-four hours of urine was collected from the animals before anaesthesia at the end of the study. The urine volume was corrected for the urinary creatinine (Cr) level, which was measured with a colorimetric method using a kit from Sigma. Calcium and phosphorus concentrations in the serum and urine were measured using commercially available 180-80-Zeeman-corrected atomic absorption spectrometer (LEEMAN-LABS, Hudson, MA, USA) according to our previous studies [9,10].

### 2.6. Serum Biochemistry

Serum samples were used for the measurement of bone-specific alkaline phosphatase (b-ALP), osteocalcin (OCN) and circulating PTH. The b-ALP concentration was determined by immunoassay (Alkphase-B, Metra Biosystems Inc., Mountain View, CA, USA). The osteocalcin content was measured using sandwich ELISA assay kit (Biomedical Technologies Inc., Stoughton, MA, USA) and PTH was measured using an ELISA kit (Immutopics Inc., San Clemente, CA, USA) according to the manufacturer’s instructions.

### 2.7. RNA Isolation and Reverse Transcription-PCR

Total RNA was extracted with Trizol reagent according to the manufacturer’s instructions. First-strand cDNA was prepared from 1 μg of total RNA using the Thermoscript reverse transcription-PCR system from Invitrogen. PTHR1 and β-actin transcripts were amplified by PCR using specific primers; PTHR1 sense: 5′-AGCGAGTGCCTCAAGTTCA-3′; anti-sense: 5′-ACAGCGTCCTTCACGAAGAT-3′; β-actin forward 5′-ATCTGGCACCACACCTTCTACAATGAGCTGCG-3′; reverse 5′-CGTCATACTCCTGCTTGCTGATCCACATCTGC-3′. PCR reactions were carried out with 1 unit of Taq polymerase in a total volume of 50 μL, and amplifications were carried out in a Peltier Thermal Cycler from MJ Research. PTHR1 amplification was done for 35 cycles (95 °C for 10 s, 60 °C for 20 s, and 72 °C for 15 s, followed extension at 72 °C for 10 min) and β-actin amplification for 20 cycles (94 °C for 35 s, 58 °C for 35 s, and 72 °C for 40 s, followed extension at 72 °C for 10 min). PCR products were analyzed on a 2% agarose gel and visualized by ethidium bromide staining.

### 2.8. Western Blot

For studying PTHR1 expression in femora tissues, femora were dissected from the animals and immediately stored in liquid nitrogen. Pooled frozen tissue was powdered and homogenized in Tris-EDTA buffer (pH 8.0). Protein concentrations were determined and equal amounts of sample were loaded on SDS-PAGE. After electrophoresis and separation, samples were transferred onto nitrocellulose membranes. Non-specific binding was blocked by incubating the membranes in 5% non-fat milk in Tris-buffered saline plus 0.1% Tween-20 (TBST) for 1 h at room temperature, followed by overnight incubation with the indicated antibodies. The immobilized PTHR1 proteins were quantitatively detected using rabbit anti-PTHR1 monoclonal antibody overnight at 4 °C followed by HRP-conjugated goat anti-rabbit IgG. β-actin was selected as an internal control. Protein bands were visualized by chemiluminescence with the enhanced chemiluminescent detection kit according to the manufacturer’s instructions.

### 2.9. Immunohistochemistry

At the end of the present study, bilateral kidneys were removed for the detection of PTHR1. The kidneys were cleaned of soft tissue and placed in fixation fluid containing 4% paraformaldehyde. After 24 h of immobilization, the kidneys were embedded with paraffin and cut into a 5 μm thick slice. Slice was mounted on a glass slide and subsequently placed in dimethyl benzene for deparaffinization. Tissue samples were therefore incubated with rabbit anti-rat PTHR1 antibody overnight at 4 °C. Slice was washed with 0.01 M PBS followed by HRP-conjugated goat anti-rabbit IgG for 2 h at room temperature. PTHR1 in kidney was visualized by DAB reaction in 0.3% hydrogen peroxide.

### 2.10. Statistical Analysis

Data are expressed as means ± SD. Differences between groups were analyzed by one-way analysis of variance (ANOVA) followed by *LSD post hoc* test using SPSS statistical software [25]. Significance was considered at * *P* < 0.05 and ** *P* < 0.01.

## 3. Results

### 3.1. Body Weights

Body weights were monitored weekly throughout the experimental period using a digital portable scale (Model XP-1500, Denver Instrument). As expected, the body weights were greater for animals in the OVX treated groups compared with the sham group. The body weights decreased after nilestriol and genistein replacement therapy in OVX rats. There were no statistically significant differences in weights observed between any of the active treatment groups (Table 1).

### 3.2. Bone Mineral Density

**Figure 2:**
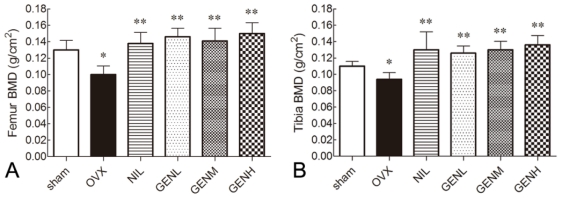
Bone mineral density (BMD) measurement of femur (**A**) and tibia (**B**). BMD significantly decreased in the animal model of ovariectomy (OVX) compared with the sham group animals. In the presence of nilestriol and genistein, BMD was recovered. Genistein dose-dependently enhanced femur and tibia BMD, and these effects were statistically significant. There were no significant difference of BMD between NIL and GEN groups. * *P* < 0.05 *vs.* sham group, ** *P* < 0.01 *vs.* OVX group.

As shown in Figure 2A, the femoral BMD was 0.130 ± 0.020 g/cm^2^ in sham group rats. Animals underwent bilateral ovariectomy revealed 23.8% reduction in BMD in the present study (*P* < 0.05). Treatment with genistein at the dose of 4.5 mg/kg (GENL), 9.0 mg/kg (GENM) and 18.0 mg/kg (GENH) appreciably recovered the loss of BMD to 0.146 ± 0.018 g/cm^2^, 0.141 ± 0.027 g/cm^2^, 0.150 ± 0.023 g/cm^2^, respectively (*P* < 0.01), in comparison with 0.100 ± 0.018 g/cm^2^ in ovariectomized animals.

We evaluated the beneficial effects of genistein treatments on tibia BMD as well (Figure 2B). The BMD of tibia in all six experimental groups showed a similar tendency as that of femur. Tibia BMD in OVX group significantly decreased to 0.094 ± 0.014 g/cm^2^ compared to 0.110 ± 0.010 g/cm^2^ of sham group. In the groups of different genistein dosages, the tibia BMD of OVX rats were all improved up to 0.126 ± 0.015 g/cm^2^, 0.130 ± 0.018 g/cm^2^ and 0.136 ± 0.020 g/cm^2^ after 12 weeks of treatments. No significant difference between nilestriol and each genistein treated groups was observed.

### 3.3. Mechanical Properties

The mean length and width of the left femur were monitored at the end of the present study. There was no significant change in length and diameter among all the experimental groups (data not shown).

**Figure 3:**
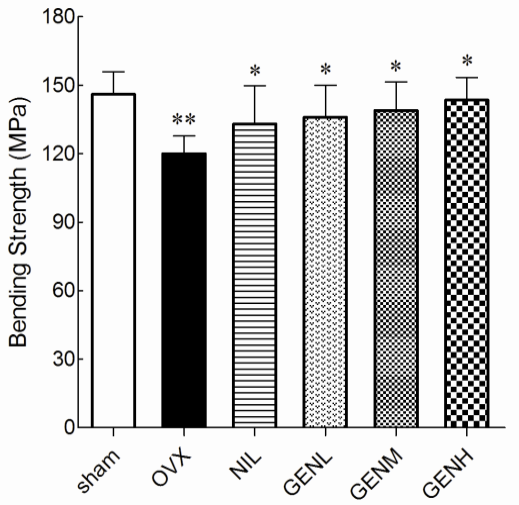
The mechanical failure properties of the femora in the sham and ovariectomized rats supplemented with nilestriol and different dose of genistein were conducted using an Instron 8501 material testing system. Femur bending strength markedly decreased after ovariectomy (** *P* < 0.01 *vs.* sham group). Treatment with nilestriol or genistein at any dosage significantly limited the failure properties that were induced by ovariectomy (* *P* < 0.05 *vs.* OVX group).

We next characterized the influence of genistein on bone mechanical properties. In the results obtained from the three-point bending test of the femur, OVX rats had significantly reduced breaking strength compared with sham group rats (*P* < 0.01). As illustrated in Figure 3, all the pharmacological treatments succeeded in improving the breaking strength of the femur (*P* < 0.05).

### 3.4. Serum and Urine Minerals and PTH Levels

To determine the genistein dosages that respectively associate to the levels of serum and urine calcium, phosphorus and circulating PTH, we further examined these minerals and hormone contents in OVX rats that were administered various doses of genistein (Table 2). Ovariectomy increased urinary calcium and phosphorus excretion, as well as decreased serum calcium and phosphorus level in ovariectomized rats, when compared to the sham group. Nilestriol treatment significantly reversed the changes of serum minerals and urinary calcium induced by ovariectomy in rats. Genistein at all doses enhanced serum mineral levels while attenuated minerals excretion in urine, and these effects were dose-dependent. Upon higher dose genistein diet feeding, the minerals restoring effects were prior to those recorded in NIL group.

Consistent with the serum calcium and phosphorus levels changes, circulating PTH in the OVX group rats was significantly lower than in the sham group. The PTH concentrations in the NIL, GENL and GENM groups animals revealed a slight increase but there were no statistically significant differences when compared with the OVX group. Genistein at a relatively high dose (18.0 mg/kg per day) raised 26.7% PTH levels in ovariectomized rats. And the difference of PTH between GENH and OVX groups was significant.

### 3.5. Effects of Genistein on Serum Bone Markers

**Figure 4:**
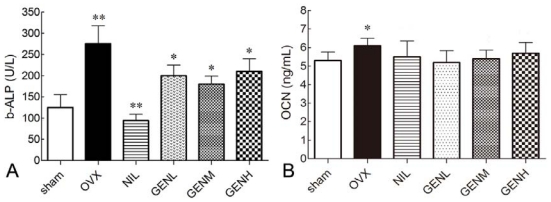
Effects of genistein on serum bone-specific alkaline phosphatase (b-ALP) and osteocalcin (OCN) concentrations in ovariectomized rats supplemented with nilestriol and different dose of genistein. (**A**) b-ALP markedly increased after ovariectomy (** *P* < 0.01 *vs.* sham group). Treatment with nilestriol (** *P* < 0.01 *vs.* OVX group) or genistein (* *P* < 0.05 *vs.* OVX group) at any dosage significantly limited b-ALP concentrations; (**B**) Serum OCN significantly increased in the animal model of ovariectomy (* *P* < 0.05 *vs.* sham group). Administration of nilestriol or genistein at any dose did not restore circulating OCN concentrations.

At the end of experiment, serum levels of b-ALP, a marker of bone formation, were 2.15-fold higher in the OVX group compared with the baseline (*P* < 0.05). The increase in b-ALP concentrations was attenuated in the presence of nilestriol. Genistein rich diet also significantly reduced serum b-ALP levels in OVX rats (*P* < 0.01). Indeed, a great dose of genistein did not cause a great reduction in serum b-ALP (Figure 4A). However, ALP levels were not restored to sham levels in the presence of NIL and different doses of GEN.

Regarding osteogenesis, circulating OCN was measured at the end of the present study as well. The serum levels of OCN were 15.1% higher in the OVX group than in the sham group (*P* < 0.05). Nilestriol at the dose of 1.5 mg/kg did not restore serum OCN concentrations. This physiological process was not prevented by a genistein diet.

### 3.6. PTHR1 Expressions in Femoral Tissues

**Figure 5:**
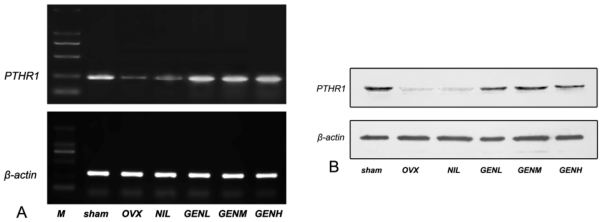
Effects of genistein treatment on parathyroid hormone receptor 1 (PTHR1) expressions in femur. (**A**) RT-PCR revealed decreased expression of PTHR1 mRNA in the animal model of ovariectomy. Nilestriol did not alleviate PTHR1 impairment. Genistein treatment recovered PTHR1 mRNA expressions in ovariectomized rats; (**B**) Western blotting analysis of femoral PTHR1 proteins showed impaired PTHR1 protein content in ovariectomized rats. Administration of nilestriol failed to reverse PTHR1 protein expression while genistein enhanced PTHR1 protein content compared with the OVX group. Genistein dose-dependently elevated both PTHR1 mRNA and protein expressions in ovariectomized rats.

To identify a PTHR isoforms, mRNA from femoral tissues was analyzed. As shown in Figure 5A, PTHR1 mRNA amplification by RT-PCR generated a fragment at 227 bp with high abundance. Expressions of β-actin mRNA were coherent among all the six groups. The ratio of PTHR1 mRNA to β-actin mRNA decreased in ovariectomized rats. Nilestriol failed to reverse PTHR1 mRNA/β-actin mRNA ratio in the animal model of ovariectomy. PTHR1 mRNA to β-actin mRNA ratio was recovered in response to genistein treatment at all dosage compared with the sham group animals. There was a tendency for a higher ratio of PTHR1 transcripts to β-actin mRNA in ovariectomized rats underwent higher dose genistein treatment. To determine the expressions of femoral PTHR1 protein in response to different treatments, Western blotting analysis of the PTHR1 protein was employed. Bilateral ovariectomy induced a reduction of PTHR1 content in femur. The impairment of PTHR1 protein did not alleviate in the presence of nilestriol. Nevertheless, genistein treatment at any dose in ovariectomized rats could enhance the expressions of femoral PTHR1 proteins compared with the OVX group animals. There was a tendency for enhanced PTHR1 protein expressions in ovariectomized rats that underwent higher dose genistein treatment as well (Figure 5B).

### 3.7. Localization and Distribution of PTHR1 in the Kidney

**Figure 6:**
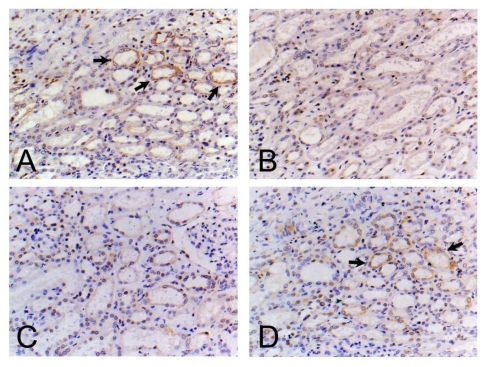
Location and distribution of PTHR1 immunoreactivity in the kidney. (**A**) sham group; (**B**) Ovariectomized (OVX) group; (**C**) OVX+ Nilestriol group (NIL); (**D**) OVX+ High dose genistein group (GENH) (18 mg/kg), respectively. The PTHR1 positive region was marked with black arrows. Original magnification ×400.

To further demonstrate whether the bone-sparing effect of genistein was mediated by PTHR1, PTHR1 immunoreactivity was studied in sections to assess the localization and distribution of the polypeptides within the kidney (Figure 6). In order to clarify effect of genistein on PTHR1 polypeptides expression, kidney samples from the GENH groups were detected. In normal female rats, PTHR1 was localized in the kidney mesenchyme. In the animal model of OVX, PTHR1 immunoreactivity was localized to a significantly lesser extent with reduced signals within kidney. However, exposure to nilestriol, an ectogenic estrogen analogue, did not alter the localization and distribution of PTHR1 after ovariectomy recovered compared with the OVX group animals. Treatment of ovariectomized rats with genistein caused significant recovery of PTHR1 expressions as evidenced by enhanced distribution and signals of PTHR1 immunoreactivity.

## 4. Discussion

The present study demonstrates that genistein treatment exerts bone-protective effect in the animal model of ovariectomy. Our major findings are summarized as follows: (1) Genistein administration improves BMD and mechanical properties in ovariectomized rats; (2) Genistein prevents post-menopausal osteoporosis by alleviating mineral loss and PTH impairments; (3) Administration of genistein limits the elevation of serum b-ALP concentration and might promote osteogenesis; (4) PTH and its receptor might participate in the process of genistein mediated osteoporotic-limiting effect.

Estrogen deficiency in post-menopausal women markedly increases the incidence of osteogenesis and osteogenesis-associated fracture and ectogenic estrogen hormone replacement therapy (HRT) has been suggested as the first-line treatment for osteogenesis in estrogen deficient women. Nevertheless, prolonged HRT is not well-tolerated due to relatively high incidence of breast, endometrial and in elevated levels of ovarian cancer risks as well as cardiovascular outcomes manifested as venous thromboembolism and stroke [4,26]. Genistein is a natural phytoestrogen derived from soy products [27]. Increasing lines of evidence suggest that genistein provides abundant clinical benefits in humans and in various animal models. Meanwhile, the genistein rich diet is well tolerated. According to Marini *et al*., the maxium genistein intake in their randomized clinical trial reached as much as 54 mg/day without signs of undesirable side effects [14]. Several studies have shown genistein-containing diet exerts estrogen-like effects in estrogen deficient animals as well as in post-menopausal women [5–8]. Other studies also highlighted genistein successfully managed vasomotor symptoms in post-menopausal women, exerted anxiolytic and anti-depressant effects in experimental animal models, revealed an excellent cardiovascular safety and positive cancer risk profile in several well controlled clinical trials [28–32]. Our previous *in vivo* studies showed short-term (4 weeks) supplements with genistein extracted from Sophora japonica–Leguminosae prevent osteoporosis in estrogen deficient rats [9]. To compare the therapeutic effects between HRT and genistein against osteogenesis, we used the animal model of bilaterally ovariectomy, which imitates accelerated bone loss due to estrogen deficient. Consistent with our previous reports, we found long-term (12 weeks) administration of commercial available genistein at three different doses prevented bone loss as well. We observed administration of genistein at all dosage could significantly preserve BMD and bending strength drop-off in ovariectomized rats.

As pointed out by Lynch *et al*., serum b-ALP and OCN concentrations have a negative correlation with osteogenesis [33]. In our experiment, limited b-ALP levels in response to genistein might indicate enhanced osteogenesis and calcium deposition. But our result on serum OCN, the most prevalent non-collagenous protein that synthesized almost exclusively by mature osteoblasts and odontoblasts in bone, showed no significant changes among all the active treated groups. It is noted ovariectomy caused a significant drop in serum minerals concentrations whereas promoted minerals losses after ovariectomy. Administration of nilestriol efficiently restored serum minerals contents and prevented minerals loss in urine. In each genistein treated groups, genistein dose-dependently restored serum minerals and inhibited minerals excretion, and these effects were statistically significant compared with the OVX group. Interestingly, we also observed a reduction of circulating PTH in ovariectomized rats. Nilestriol failed to inhibit the drop in circulating PTH concentration while ovariectomized rats supplemented with genistein showed a tendency for an increase in PTH concentrations. Based on the established effect of genistein against osteogenesis, our data suggested a potential cross-talk between genistein and PTH/PTHR1.

For these reasons, correlation between genistein and PTH/PTHR1 were analyzed to shed light on the new mechanisms of osteogenesis-limiting events initiated by genistein. RT-PCR and Western blot results showed ovariectomy impaired PTHR1 expressions at mRNA and protein levels in the femoral tissue in comparison with the baseline. PTHR1 mRNA and protein aberrations were not ameliorated after nilestriol therapy. Conversely, restored PTHR1 mRNA and protein expressions were observed in femoral tissue in ovariectomized rats supplied with different doses of genistein. Kidney specimens from different groups were studied for the location and distribution pattern of PTHR1 immunoreactivities as well. In agreement with RT-PCR and Western blot findings, visualized PTHR1 immunoreactivity by PTHR1 specific monoclonal antibody showed a reduction in the location and distribution of PTHR1 after ovariectomy. Nilestriol failed to counteract PTHR1 turnover while genistein extend PTHR1 immunoreactive location and distributions in ovariectomized rats. These results might explain the prominent profile of genistein on restoring serum minerals while alleviating mineral excretion in the urine. Herein, it is reasonable to believe the anti-osteoporotic effect of genistein is partly PTH/PTHR1 dependent.

## 5. Conclusion

In summary, the present study highlights beneficial effects of genistein in the treatment of ovariectomy-induced osteoporosis. Genistein restores bending strength, BMD and minerals together with serum bone markers in ovariectomized rats. Furthermore, genistein alters circulating PTH concentration and PTHR1 expressions in bone as well as in the kidney. Our study reports for the first time that genistein administration could modulate PTH/PTHR1 expressions at mRNA and protein levels in ovariectomized animals. Apart from acting as an estrogen analogue, genistein might interact with PTH/PTHR1 during ovariectomy-induced osteoporosis. This novel finding suggests the bone-sparing effect of genistein in estrogen deficient rats is partly PTH/PTHR1-dependent. Therefore, genistein might be a potential option in the therapeutic strategy of post-menopausal osteoporosis with good tolerance and few undesirable side effects.

## Figures and Tables

**Table 1:** Body weight (g) in the sham and ovariectomized rats supplemented with nilestriol and different dose of genistein.

	0 week	1 week	2 weeks	4 weeks	8 weeks	12 weeks
sham	229.6 ± 7.1	248.3 ± 9.7	242.5 ± 10.3	252.8 ± 14.8	303.3 ± 23.7	324.3 ± 35.2
OVX	223.4 ± 19.1	254.7 ± 28.2	273.4 ± 15.3 a	298.0 ± 32.6 a	310.7 ± 23.0	325.3 ± 31.7
NIL	226.3 ± 15.3	246.2 ± 12.9	250.1 ± 4.8 b	264.9 ± 21.9 b	277.2 ± 14.7 b	297.1 ± 27.5 b
GENL	228.7 ± 11.2	250.9 ± 10.3	265.2 ± 25.3	276.1 ± 24.2 b	280.7 ± 35.9 b	312.8 ± 26.7
GENM	225.5 ± 13.3	262.2 ± 13.4	268.0 ± 14.7	284.6 ± 17.5 b	329.7 ± 20.5 b	347.7 ± 31.2 b
GENH	227.4 ± 9.8	255.0 ± 18.1	261.4 ± 8.7	275.2 ± 10.5 b	312.1 ± 22.4	328.8 ± 36.3

*n* = 20,

a *P* < 0.05 *vs.* sham;

b *P* < 0.05 *vs.* OVX, respectively.

**Table 2:** Serum and urine minerals and circulating parathyroid hormone (PTH) concentrations in the sham and ovariectomized rats supplemented with nilestriol and different dose of genistein.

Group	Serum Ca (mmol/L)	Serum P (mmol/L)	Urine Ca/Cr	Urine P/Cr	PTH (pg/mL)
Sham	2.15 ± 0.53	2.39 ± 0.24	0.30 ± 0.03	4.47 ± 0.23	108.54 ± 12.36
OVX	1.72 ± 0.36 a	2.00 ± 0.39 a	0.58 ± 0.02 a	5.85 ± 0.30 a	5.14 ± 17.49 a
NIL	1.90 ± 0.63 b	2.21 ± 0.32 b	0.35 ± 0.01 b	4.82 ± 0.43 b	83.43 ± 20.78
GENL	1.83 ± 0.40 b	2.45 ± 0.38 b	0.49 ± 0.06 b	5.48 ± 0.14 b	78.62 ± 18.95
GENM	2.09 ± 0.31 b	2.63 ± 0.84 b	0.37 ± 0.05 b	5.00 ± 0.36 b	80.23 ± 22.54
GENH	2.12 ± 0.42 b	2.71 ± 0.75 b	0.32 ± 0.02 b	4.64 ± 0.20 b	95.37 ± 21.57 b

*n* = 20,

a *P* < 0.05 *vs.* sham;

b *P* < 0.05 *vs.* OVX, respectively.
